# Supplementary material for: OpenMM 7: Rapid development of high performance algorithms for molecular dynamics
Source: PLoS Comput Biol. 2017 Jul 26;13(7):e1005659. doi: 10.1371/journal.pcbi.1005659 (PMC5549999; doi:10.1371/journal.pcbi.1005659)
Supplement: S1 Source Code — (ZIP) [file pcbi.1005659.s002.zip › OpenMM-7.0.1-Source/docs/api-c++/generated/OpenMM.CustomGBForce.html]

CustomGBForce — OpenMM @OPENMM\_MAJOR\_VERSION@.@OPENMM\_MINOR\_VERSION@ documentation


# CustomGBForce¶

*class* `OpenMM::``CustomGBForce`¶
:   This class implements complex, multiple stage nonbonded interactions between particles. It is designed primarily for implementing Generalized Born implicit solvation models, although it is not strictly limited to that purpose. The interaction is specified as a series of computations, each defined by an arbitrary algebraic expression. It also allows tabulated functions to be defined and used with the computations. It optionally supports periodic boundary conditions and cutoffs for long range interactions.

    The computation consists of calculating some number of per-particle

    When specifying a computed value or energy term, you provide an algebraic expression to evaluate and a

    > - `System`. In the case of a computed value, this means the value for a particle depends only on other properties of that particle (its position, parameters, and other computed values). In the case of an energy term, it means each particle makes an independent contribution to the `System` energy.
    > - `System` energy. (Note that energy terms are assumed to be symmetric with respect to the two interacting particles, and therefore are evaluated only once per pair. In contrast, expressions for computed values need not be symmetric and therefore are calculated twice for each pair: once when calculating the value for the first particle, and again when calculating the value for the second particle.)

    Be aware that, although this class is extremely general in the computations it can define, particular Platforms may only support more restricted types of computations. In particular, all currently existing Platforms require that the first computed value

    This is a complicated class to use, and an example may help to clarify it. The following code implements the OBC variant of the GB/SA solvation model, using the ACE approximation to estimate surface area:

    ```
    CustomGBForce* custom = new CustomGBForce();
    custom->addPerParticleParameter("q");
    custom->addPerParticleParameter("radius");
    custom->addPerParticleParameter("scale");
    custom->addGlobalParameter("solventDielectric", obc->getSolventDielectric());
    custom->addGlobalParameter("soluteDielectric", obc->getSoluteDielectric());
    custom->addComputedValue("I", "step(r+sr2-or1)*0.5*(1/L-1/U+0.25*(1/U^2-1/L^2)*(r-sr2*sr2/r)+0.5*log(L/U)/r+C);"
                                  "U=r+sr2;"
                                  "C=2*(1/or1-1/L)*step(sr2-r-or1);"
                                  "L=max(or1, D);"
                                  "D=abs(r-sr2);"
                                  "sr2 = scale2*or2;"
                                  "or1 = radius1-0.009; or2 = radius2-0.009", CustomGBForce::ParticlePairNoExclusions);
    custom->addComputedValue("B", "1/(1/or-tanh(1*psi-0.8*psi^2+4.85*psi^3)/radius);"
                                  "psi=I*or; or=radius-0.009", CustomGBForce::SingleParticle);
    custom->addEnergyTerm("28.3919551*(radius+0.14)^2*(radius/B)^6-0.5*138.935456*(1/soluteDielectric-1/solventDielectric)*q^2/B",
                          CustomGBForce::SingleParticle);
    custom->addEnergyTerm("-138.935456*(1/soluteDielectric-1/solventDielectric)*q1*q2/f;"
                          "f=sqrt(r^2+B1*B2*exp(-r^2/(4*B1*B2)))", CustomGBForce::ParticlePair);
    ```

    It begins by defining three per-particle parameters (charge, atomic radius, and scale factor) and two global parameters (the dielectric constants for the solute and solvent). It then defines a computed value “I” of type ParticlePair. The expression for evaluating it is a complicated function of the distance between each pair of particles (r), their atomic radii (radius1 and radius2), and their scale factors (scale1 and scale2). Very roughly speaking, it is a measure of the distance between each particle and other nearby particles.

    Next a computation is defined for the Born Radius (B). It is computed independently for each particle, and is a function of that particle’s atomic radius and the intermediate value I defined above.

    Finally, two energy terms are defined. The first one is computed for each particle and represents the surface area term, as well as the self interaction part of the polarization energy. The second term is calculated for each pair of particles, and represents the screening of electrostatic interactions by the solvent.

    After defining the force as shown above, you should then call `addParticle()` once for each particle in the `System` to set the values of its per-particle parameters (q, radius, and scale). The number of particles for which you set parameters must be exactly equal to the number of particles in the `System`, or else an exception will be thrown when you try to create a `Context`. After a particle has been added, you can modify its parameters by calling `setParticleParameters()`. This will have no effect on Contexts that already exist unless you call `updateParametersInContext()`.
    `CustomGBForce` also lets you specify “exclusions”, particular pairs of particles whose interactions should be omitted from calculations. This is most often used for particles that are bonded to each other. Even if you specify exclusions, however, you can use the computation type ParticlePairNoExclusions to indicate that exclusions should not be applied to a particular piece of the computation.

    Expressions may involve the operators + (add), - (subtract), \* (multiply), / (divide), and ^ (power), and the following functions: sqrt, exp, log, sin, cos, sec, csc, tan, cot, asin, acos, atan, sinh, cosh, tanh, erf, erfc, min, max, abs, floor, ceil, step, delta, select. All trigonometric functions are defined in radians, and log is the natural logarithm. step(x) = 0 if x is less than 0, 1 otherwise. delta(x) = 1 if x is 0, 0 otherwise. select(x,y,z) = z if x = 0, y otherwise. In expressions for particle pair calculations, the names of per-particle parameters and computed values have the suffix “1” or “2” appended to them to indicate the values for the two interacting particles. As seen in the above example, an expression may also involve intermediate quantities that are defined following the main expression, using ”;” as a separator.

    In addition, you can call `addTabulatedFunction()` to define a new function based on tabulated values. You specify the function by creating a `TabulatedFunction` object. That function can then appear in expressions.

    Methods

    |  |  |
    | --- | --- |
    | `CustomGBForce` | Create a `CustomGBForce`. |
    | `~CustomGBForce` |  |
    | `getNumParticles` | Get the number of particles for which force field parameters have been defined. |
    | `getNumExclusions` | Get the number of particle pairs whose interactions should be excluded. |
    | `getNumPerParticleParameters` | Get the number of per-particle parameters that the interaction depends on. |
    | `getNumGlobalParameters` | Get the number of global parameters that the interaction depends on. |
    | `getNumTabulatedFunctions` | Get the number of tabulated functions that have been defined. |
    | `getNumFunctions` | Get the number of tabulated functions that have been defined. |
    | `getNumComputedValues` | Get the number of per-particle computed values the interaction depends on. |
    | `getNumEnergyTerms` | Get the number of terms in the energy computation. |
    | `getNonbondedMethod` | Get the method used for handling long range nonbonded interactions. |
    | `setNonbondedMethod` | Set the method used for handling long range nonbonded interactions. |
    | `getCutoffDistance` | Get the cutoff distance (in nm) being used for nonbonded interactions. |
    | `setCutoffDistance` | Set the cutoff distance (in nm) being used for nonbonded interactions. |
    | `addPerParticleParameter` | Add a new per-particle parameter that the interaction may depend on. |
    | `getPerParticleParameterName` | Get the name of a per-particle parameter. |
    | `setPerParticleParameterName` | Set the name of a per-particle parameter. |
    | `addGlobalParameter` | Add a new global parameter that the interaction may depend on. |
    | `getGlobalParameterName` | Get the name of a global parameter. |
    | `setGlobalParameterName` | Set the name of a global parameter. |
    | `getGlobalParameterDefaultValue` | Get the default value of a global parameter. |
    | `setGlobalParameterDefaultValue` | Set the default value of a global parameter. |
    | `addParticle` | Add the nonbonded force parameters for a particle. |
    | `getParticleParameters` | Get the nonbonded force parameters for a particle. |
    | `setParticleParameters` | Set the nonbonded force parameters for a particle. |
    | `addComputedValue` | Add a computed value to calculate for each particle. |
    | `getComputedValueParameters` | Get the properties of a computed value. |
    | `setComputedValueParameters` | Set the properties of a computed value. |
    | `addEnergyTerm` | Add a term to the energy computation. |
    | `getEnergyTermParameters` | Get the properties of a term to the energy computation. |
    | `setEnergyTermParameters` | Set the properties of a term to the energy computation. |
    | `addExclusion` | Add a particle pair to the list of interactions that should be excluded. |
    | `getExclusionParticles` | Get the particles in a pair whose interaction should be excluded. |
    | `setExclusionParticles` | Set the particles in a pair whose interaction should be excluded. |
    | `addTabulatedFunction` | Add a tabulated function that may appear in expressions. |
    | `getTabulatedFunction` | Get a const reference to a tabulated function that may appear in expressions. |
    | `getTabulatedFunction` | Get a reference to a tabulated function that may appear in expressions. |
    | `getTabulatedFunctionName` | Get the name of a tabulated function that may appear in expressions. |
    | `addFunction` | Add a tabulated function that may appear in expressions. |
    | `getFunctionParameters` | Get the parameters for a tabulated function that may appear in expressions. |
    | `setFunctionParameters` | Set the parameters for a tabulated function that may appear in expressions. |
    | `updateParametersInContext` | Update the per-particle parameters in a `Context` to match those stored in this `Force` object. |
    | `usesPeriodicBoundaryConditions` | Returns whether or not this force makes use of periodic boundary conditions. |

    Enum: NonbondedMethod

    |  |  |
    | --- | --- |
    | **NoCutoff** | No cutoff is applied to nonbonded interactions. The full set of N^2 interactions is computed exactly. This necessarily means that periodic boundary conditions cannot be used. This is the default. |
    | **CutoffNonPeriodic** | Interactions beyond the cutoff distance are ignored. |
    | **CutoffPeriodic** | Periodic boundary conditions are used, so that each particle interacts only with the nearest periodic copy of each other particle. Interactions beyond the cutoff distance are ignored. |

    Enum: ComputationType

    |  |  |
    | --- | --- |
    | **SingleParticle** | The value is computed independently for each particle, based only on the parameters and computed values for that particle. |
    | **ParticlePair** | The value is computed as a sum over all pairs of particles, except those which have been added as exclusions. |
    | **ParticlePairNoExclusions** | The value is computed as a sum over all pairs of particles. Unlike ParticlePair, the list of exclusions is ignored and all pairs are included in the sum, even those marked as exclusions. |

    `CustomGBForce`()¶
    :   Create a `CustomGBForce`.

    `~CustomGBForce`()¶

    int `getNumParticles`() *const*¶
    :   Get the number of particles for which force field parameters have been defined.

    int `getNumExclusions`() *const*¶
    :   Get the number of particle pairs whose interactions should be excluded.

    int `getNumPerParticleParameters`() *const*¶
    :   Get the number of per-particle parameters that the interaction depends on.

    int `getNumGlobalParameters`() *const*¶
    :   Get the number of global parameters that the interaction depends on.

    int `getNumTabulatedFunctions`() *const*¶
    :   Get the number of tabulated functions that have been defined.

    int `getNumFunctions`() *const*¶
    :   Get the number of tabulated functions that have been defined.

        Deprecated

        This method exists only for backward compatibility. Use `getNumTabulatedFunctions()` instead.

    int `getNumComputedValues`() *const*¶
    :   Get the number of per-particle computed values the interaction depends on.

    int `getNumEnergyTerms`() *const*¶
    :   Get the number of terms in the energy computation.

    NonbondedMethod `getNonbondedMethod`() *const*¶
    :   Get the method used for handling long range nonbonded interactions.

    void `setNonbondedMethod`(NonbondedMethod *method*)¶
    :   Set the method used for handling long range nonbonded interactions.

    double `getCutoffDistance`() *const*¶
    :   Get the cutoff distance (in nm) being used for nonbonded interactions. If the NonbondedMethod in use is NoCutoff, this value will have no effect.

        |  |  |
        | --- | --- |
        | Returns: | the cutoff distance, measured in nm |

    void `setCutoffDistance`(double *distance*)¶
    :   Set the cutoff distance (in nm) being used for nonbonded interactions. If the NonbondedMethod in use is NoCutoff, this value will have no effect.

        |  |  |
        | --- | --- |
        | Parameters: |  |

        - **distance** – the cutoff distance, measured in nm

    int `addPerParticleParameter`(*const* std::string &*name*)¶
    :   Add a new per-particle parameter that the interaction may depend on.

        |  |  |
        | --- | --- |
        | Parameters: |  |

        - **name** – the name of the parameter

        |  |  |
        | --- | --- |
        | Returns: | the index of the parameter that was added |

    *const* std::string &`getPerParticleParameterName`(int *index*) *const*¶
    :   Get the name of a per-particle parameter.

        |  |  |
        | --- | --- |
        | Parameters: |  |

        - **index** – the index of the parameter for which to get the name

        |  |  |
        | --- | --- |
        | Returns: | the parameter name |

    void `setPerParticleParameterName`(int *index*, *const* std::string &*name*)¶
    :   Set the name of a per-particle parameter.

        |  |  |
        | --- | --- |
        | Parameters: |  |

        - **index** – the index of the parameter for which to set the name
        - **name** – the name of the parameter

    int `addGlobalParameter`(*const* std::string &*name*, double *defaultValue*)¶
    :   Add a new global parameter that the interaction may depend on.

        |  |  |
        | --- | --- |
        | Parameters: |  |

        - **name** – the name of the parameter
        - **defaultValue** – the default value of the parameter

        |  |  |
        | --- | --- |
        | Returns: | the index of the parameter that was added |

    *const* std::string &`getGlobalParameterName`(int *index*) *const*¶
    :   Get the name of a global parameter.

        |  |  |
        | --- | --- |
        | Parameters: |  |

        - **index** – the index of the parameter for which to get the name

        |  |  |
        | --- | --- |
        | Returns: | the parameter name |

    void `setGlobalParameterName`(int *index*, *const* std::string &*name*)¶
    :   Set the name of a global parameter.

        |  |  |
        | --- | --- |
        | Parameters: |  |

        - **index** – the index of the parameter for which to set the name
        - **name** – the name of the parameter

    double `getGlobalParameterDefaultValue`(int *index*) *const*¶
    :   Get the default value of a global parameter.

        |  |  |
        | --- | --- |
        | Parameters: |  |

        - **index** – the index of the parameter for which to get the default value

        |  |  |
        | --- | --- |
        | Returns: | the parameter default value |

    void `setGlobalParameterDefaultValue`(int *index*, double *defaultValue*)¶
    :   Set the default value of a global parameter.

        |  |  |
        | --- | --- |
        | Parameters: |  |

        - **index** – the index of the parameter for which to set the default value
        - **defaultValue** – the default value of the parameter

    int `addParticle`(*const* std::vector<double> &*parameters* = std::vector< double >())¶
    :   Add the nonbonded force parameters for a particle. This should be called once for each particle in the `System`. When it is called for the i’th time, it specifies the parameters for the i’th particle.

        |  |  |
        | --- | --- |
        | Parameters: |  |

        - **parameters** – the list of parameters for the new particle

        |  |  |
        | --- | --- |
        | Returns: | the index of the particle that was added |

    void `getParticleParameters`(int *index*, std::vector<double> &*parameters*) *const*¶
    :   Get the nonbonded force parameters for a particle.

        |  |  |
        | --- | --- |
        | Parameters: |  |

        - **index** – the index of the particle for which to get parameters
        - **parameters** – [out] the list of parameters for the specified particle

    void `setParticleParameters`(int *index*, *const* std::vector<double> &*parameters*)¶
    :   Set the nonbonded force parameters for a particle.

        |  |  |
        | --- | --- |
        | Parameters: |  |

        - **index** – the index of the particle for which to set parameters
        - **parameters** – the list of parameters for the specified particle

    int `addComputedValue`(*const* std::string &*name*, *const* std::string &*expression*, ComputationType *type*)¶
    :   Add a computed value to calculate for each particle.

        |  |  |
        | --- | --- |
        | Parameters: |  |

        - **name** – the name of the value
        - **expression** – an algebraic expression to evaluate when calculating the computed value. If the ComputationType is SingleParticle, the expression is evaluated independently for each particle, and may depend on its x, y, and z coordinates, as well as the per-particle parameters and previous computed values for that particle. If the ComputationType is ParticlePair or ParticlePairNoExclusions, the expression is evaluated once for every other particle in the system and summed to get the final value. In the latter case, the expression may depend on the distance r between the two particles, and on the per-particle parameters and previous computed values for each of them. Append “1” to a variable name to indicate the parameter for the particle whose value is being calculated, and “2” to indicate the particle it is interacting with.
        - **type** – the method to use for computing this value

    void `getComputedValueParameters`(int *index*, std::string &*name*, std::string &*expression*, ComputationType &*type*) *const*¶
    :   Get the properties of a computed value.

        |  |  |
        | --- | --- |
        | Parameters: |  |

        - **index** – the index of the computed value for which to get parameters
        - **name** – [out] the name of the value
        - **expression** – [out] an algebraic expression to evaluate when calculating the computed value. If the ComputationType is SingleParticle, the expression is evaluated independently for each particle, and may depend on its x, y, and z coordinates, as well as the per-particle parameters and previous computed values for that particle. If the ComputationType is ParticlePair or ParticlePairNoExclusions, the expression is evaluated once for every other particle in the system and summed to get the final value. In the latter case, the expression may depend on the distance r between the two particles, and on the per-particle parameters and previous computed values for each of them. Append “1” to a variable name to indicate the parameter for the particle whose value is being calculated, and “2” to indicate the particle it is interacting with.
        - **type** – [out] the method to use for computing this value

    void `setComputedValueParameters`(int *index*, *const* std::string &*name*, *const* std::string &*expression*, ComputationType *type*)¶
    :   Set the properties of a computed value.

        |  |  |
        | --- | --- |
        | Parameters: |  |

        - **index** – the index of the computed value for which to set parameters
        - **name** – the name of the value
        - **expression** – an algebraic expression to evaluate when calculating the computed value. If the ComputationType is SingleParticle, the expression is evaluated independently for each particle, and may depend on its x, y, and z coordinates, as well as the per-particle parameters and previous computed values for that particle. If the ComputationType is ParticlePair or ParticlePairNoExclusions, the expression is evaluated once for every other particle in the system and summed to get the final value. In the latter case, the expression may depend on the distance r between the two particles, and on the per-particle parameters and previous computed values for each of them. Append “1” to a variable name to indicate the parameter for the particle whose value is being calculated, and “2” to indicate the particle it is interacting with.
        - **type** – the method to use for computing this value

    int `addEnergyTerm`(*const* std::string &*expression*, ComputationType *type*)¶
    :   Add a term to the energy computation.

        |  |  |
        | --- | --- |
        | Parameters: |  |

        - **expression** – an algebraic expression to evaluate when calculating the energy. If the ComputationType is SingleParticle, the expression is evaluated once for each particle, and may depend on its x, y, and z coordinates, as well as the per-particle parameters and computed values for that particle. If the ComputationType is ParticlePair or ParticlePairNoExclusions, the expression is evaluated once for every pair of particles in the system. In the latter case, the expression may depend on the distance r between the two particles, and on the per-particle parameters and computed values for each of them. Append “1” to a variable name to indicate the parameter for the first particle in the pair and “2” to indicate the second particle in the pair.
        - **type** – the method to use for computing this value

    void `getEnergyTermParameters`(int *index*, std::string &*expression*, ComputationType &*type*) *const*¶
    :   Get the properties of a term to the energy computation.

        |  |  |
        | --- | --- |
        | Parameters: |  |

        - **index** – the index of the term for which to get parameters
        - **expression** – [out] an algebraic expression to evaluate when calculating the energy. If the ComputationType is SingleParticle, the expression is evaluated once for each particle, and may depend on its x, y, and z coordinates, as well as the per-particle parameters and computed values for that particle. If the ComputationType is ParticlePair or ParticlePairNoExclusions, the expression is evaluated once for every pair of particles in the system. In the latter case, the expression may depend on the distance r between the two particles, and on the per-particle parameters and computed values for each of them. Append “1” to a variable name to indicate the parameter for the first particle in the pair and “2” to indicate the second particle in the pair.
        - **type** – [out] the method to use for computing this value

    void `setEnergyTermParameters`(int *index*, *const* std::string &*expression*, ComputationType *type*)¶
    :   Set the properties of a term to the energy computation.

        |  |  |
        | --- | --- |
        | Parameters: |  |

        - **index** – the index of the term for which to set parameters
        - **expression** – an algebraic expression to evaluate when calculating the energy. If the ComputationType is SingleParticle, the expression is evaluated once for each particle, and may depend on its x, y, and z coordinates, as well as the per-particle parameters and computed values for that particle. If the ComputationType is ParticlePair or ParticlePairNoExclusions, the expression is evaluated once for every pair of particles in the system. In the latter case, the expression may depend on the distance r between the two particles, and on the per-particle parameters and computed values for each of them. Append “1” to a variable name to indicate the parameter for the first particle in the pair and “2” to indicate the second particle in the pair.
        - **type** – the method to use for computing this value

    int `addExclusion`(int *particle1*, int *particle2*)¶
    :   Add a particle pair to the list of interactions that should be excluded.

        |  |  |
        | --- | --- |
        | Parameters: |  |

        - **particle1** – the index of the first particle in the pair
        - **particle2** – the index of the second particle in the pair

        |  |  |
        | --- | --- |
        | Returns: | the index of the exclusion that was added |

    void `getExclusionParticles`(int *index*, int &*particle1*, int &*particle2*) *const*¶
    :   Get the particles in a pair whose interaction should be excluded.

        |  |  |
        | --- | --- |
        | Parameters: |  |

        - **index** – the index of the exclusion for which to get particle indices
        - **particle1** – [out] the index of the first particle in the pair
        - **particle2** – [out] the index of the second particle in the pair

    void `setExclusionParticles`(int *index*, int *particle1*, int *particle2*)¶
    :   Set the particles in a pair whose interaction should be excluded.

        |  |  |
        | --- | --- |
        | Parameters: |  |

        - **index** – the index of the exclusion for which to set particle indices
        - **particle1** – the index of the first particle in the pair
        - **particle2** – the index of the second particle in the pair

    int `addTabulatedFunction`(*const* std::string &*name*, TabulatedFunction \**function*)¶
    :   Add a tabulated function that may appear in expressions.

        |  |  |
        | --- | --- |
        | Parameters: |  |

        - **name** – the name of the function as it appears in expressions
        - **function** – a `TabulatedFunction` object defining the function. The `TabulatedFunction` should have been created on the heap with the “new” operator. The `Force` takes over ownership of it, and deletes it when the `Force` itself is deleted.

        |  |  |
        | --- | --- |
        | Returns: | the index of the function that was added |

    *const* TabulatedFunction &`getTabulatedFunction`(int *index*) *const*¶
    :   Get a const reference to a tabulated function that may appear in expressions.

        |  |  |
        | --- | --- |
        | Parameters: |  |

        - **index** – the index of the function to get

        |  |  |
        | --- | --- |
        | Returns: | the `TabulatedFunction` object defining the function |

    TabulatedFunction &`getTabulatedFunction`(int *index*)¶
    :   Get a reference to a tabulated function that may appear in expressions.

        |  |  |
        | --- | --- |
        | Parameters: |  |

        - **index** – the index of the function to get

        |  |  |
        | --- | --- |
        | Returns: | the `TabulatedFunction` object defining the function |

    *const* std::string &`getTabulatedFunctionName`(int *index*) *const*¶
    :   Get the name of a tabulated function that may appear in expressions.

        |  |  |
        | --- | --- |
        | Parameters: |  |

        - **index** – the index of the function to get

        |  |  |
        | --- | --- |
        | Returns: | the name of the function as it appears in expressions |

    int `addFunction`(*const* std::string &*name*, *const* std::vector<double> &*values*, double *min*, double *max*)¶
    :   Add a tabulated function that may appear in expressions.

        Deprecated

        This method exists only for backward compatibility. Use `addTabulatedFunction()` instead.

    void `getFunctionParameters`(int *index*, std::string &*name*, std::vector<double> &*values*, double &*min*, double &*max*) *const*¶
    :   Get the parameters for a tabulated function that may appear in expressions.

        Deprecated

        This method exists only for backward compatibility. Use getTabulatedFunctionParameters() instead. If the specified function is not a `Continuous1DFunction`, this throws an exception.

    void `setFunctionParameters`(int *index*, *const* std::string &*name*, *const* std::vector<double> &*values*, double *min*, double *max*)¶
    :   Set the parameters for a tabulated function that may appear in expressions.

        Deprecated

        This method exists only for backward compatibility. Use setTabulatedFunctionParameters() instead. If the specified function is not a `Continuous1DFunction`, this throws an exception.

    void `updateParametersInContext`(Context &*context*)¶
    :   Update the per-particle parameters in a `Context` to match those stored in this `Force` object. This method provides an efficient method to update certain parameters in an existing `Context` without needing to reinitialize it. Simply call `setParticleParameters()` to modify this object’s parameters, then call `updateParametersInContext()` to copy them over to the `Context`.

        This method has several limitations. The only information it updates is the values of per-particle parameters. All other aspects of the `Force` (such as the energy function) are unaffected and can only be changed by reinitializing the `Context`. Also, this method cannot be used to add new particles, only to change the parameters of existing ones.

    bool `usesPeriodicBoundaryConditions`() *const*¶
    :   Returns whether or not this force makes use of periodic boundary conditions.

        |  |  |
        | --- | --- |
        | Returns: | true if force uses PBC and false otherwise |

High performance molecular simulation on GPUs


### Navigation

- Core Objects
- Forces
- Integrators
- Extras

©2015, Stanford University and the Authors.
|
Powered by Sphinx 1.3.1
& Alabaster 0.7.7
|
Page source
